# Human adipose stromal cell therapy improves survival and reduces renal inflammation and capillary rarefaction in acute kidney injury

**DOI:** 10.1111/jcmm.13071

**Published:** 2017-04-28

**Authors:** Jason A. Collett, Dmitry O. Traktuev, Purvi Mehrotra, Allison Crone, Stephanie Merfeld‐Clauss, Keith L. March, David P. Basile

**Affiliations:** ^1^ Department of Cellular and Integrative PhysiologyKrannert Institute of CardiologyIndiana University School of MedicineIndiana Center for Vascular Biology and MedicineIndianapolisINUSA; ^2^ VA Center for Regenerative Medicine IndianapolisRichard L. Roudebush VA Medical CenterIndianapolisINUSA; ^3^ Department of MedicineKrannert Institute of CardiologyIndiana University School of MedicineIndiana Center for Vascular Biology and MedicineIndianapolisINUSA

**Keywords:** adipose stem cells, stromal cells, acute kidney injury, inflammation

## Abstract

Damage to endothelial cells contributes to acute kidney injury (AKI) by causing impaired perfusion, while the permanent loss of the capillary network following AKI has been suggested to promote chronic kidney disease. Therefore, strategies to protect renal vasculature may impact both short‐term recovery and long‐term functional preservation post‐AKI. Human adipose stromal cells (hASCs) possess pro‐angiogenic and anti‐inflammatory properties and therefore have been tested as a therapeutic agent to treat ischaemic conditions. This study evaluated hASC potential to facilitate recovery from AKI with specific attention to capillary preservation and inflammation. Male Sprague Dawley rats were subjected to bilateral ischaemia/reperfusion and allowed to recover for either two or seven days. At the time of reperfusion, hASCs or vehicle was injected into the suprarenal abdominal aorta. hASC‐treated rats had significantly greater survival compared to vehicle‐treated rats (88.7% *versus* 69.3%). hASC treatment showed hastened recovery as demonstrated by lower creatinine levels at 48 hrs, while tubular damage was significantly reduced at 48 hrs. hASC treatment resulted in a significant decrease in total T cell and Th17 cell infiltration into injured kidneys at 2 days post‐AKI, but an increase in accumulation of regulatory T cells. By day 7, hASC‐treated rats showed significantly attenuated capillary rarefaction in the cortex (15% *versus* 5%) and outer medulla (36% *versus* 18%) compared to vehicle‐treated rats as well as reduced accumulation of interstitial alpha‐smooth muscle actin‐positive myofibroblasts. These results suggest for the first time that hASCs improve recovery from I/R‐induced injury by mechanisms that contribute to decrease in inflammation and preservation of peritubular capillaries.

## Introduction

Acute kidney injury (AKI) is associated with a high mortality rate, is the leading cause of nephrology consultation and results in costs to the healthcare system of approximately $10 billion annually 1. Although there are many causes of AKI, decreased renal perfusion secondary to endothelial damage represents a leading cause contributing to kidney injury and loss of renal function 2. Mounting evidence suggests that AKI can contribute to chronic kidney disease (CKD) and enhances the risk of end‐stage renal disease (ESRD) 3, 4. The link between AKI and the development of CKD likely involves alterations in renal structure following tissue repair that predispose the development of interstitial fibrosis. For example, despite recovery of serum creatinine, it has been shown that renal capillary density is permanently reduced in rats following AKI 5, 6. It has been suggested that this capillary rarefaction exacerbates renal hypoxia and accelerates interstitial fibrosis 7, 8, 9. In this regard, strategies that improve renal blood flow or preserve renal capillaries have been shown to provide better long‐term protection from CKD development 7, 8, 9. In parallel, it is clear that activation of immune cells in post‐ischaemic kidneys also plays an important role in the progression of CKD following AKI 10, 11. These observations emphasize that therapeutic approaches that target both endothelial and inflammatory processes may result in better short‐term structural recovery and mitigate progression of chronic pathological conditions.

Stem cell‐based approaches have been tested as potential therapies in the setting of AKI, as they offer the opportunity to promote tissue repair. While studies performed with bone marrow‐derived haematopoietic progenitors have proved disappointing, marrow‐derived mesenchymal stem cells (MSCs) have shown promise in the treatment of various ischaemic insults, including AKI 12, 13, 14, 15. However, the vascular protective effects and potential to mitigate progression to CKD following AKI have thus far received little attention.

Adipose‐derived stromal cells (ASCs) are multipotent mesenchymal progenitor cells that functionally and phenotypically resemble pericytes, with the ability to stabilize endothelial networks 16. While retaining many properties of marrow‐derived MSCs, ASCs may be advantageous as they are easier to isolate, highly expandable and therefore are more feasible for translation into the clinic. It has been well accepted that the primary mechanism of activity of systemically infused therapeutic cells is their paracrine activity. Multiple studies have shown that ASCs produce a variety of soluble cytokines and exosomes that have anti‐apoptotic, angiogenic, anti‐fibrotic and immunomodulatory activities 17, 18, 19, 20, 21. We and others have shown that these cell properties allow ASCs to be used to treat multiple pathological conditions associated with acute local ischaemia (*e.g*. myocardial infarction) and inflammation 22, 23, 24, 25. Other investigators have shown that ASCs may also protect against ischaemic AKI 21, 26, 27, 28, but these studies utilized mouse‐derived ASCs and did not examine effects of cells on vascular preservation. To determine whether human ASC‐based therapies may represent a potential treatment for AKI, the current study was conducted using a well‐established rat model of AKI induced by I/R injury, with a particular focus on hASC effects on renal vasculature preservation as well as lymphocyte activation, as these two features are known to be associated with progression of CKD following the initial recovery from AKI.

## Materials and methods

### Animals and cells

Male Sprague Dawley rats (250–300 g) were purchased from Harlan (Indianapolis, IN, USA). Rats were maintained in accordance with the policies of the National Institutes of Health *Guide for the Care and Use of Laboratory Animals*. All protocols were approved by Institutional Animal Care and Use Committee at Indiana University.

**Figure 1:**
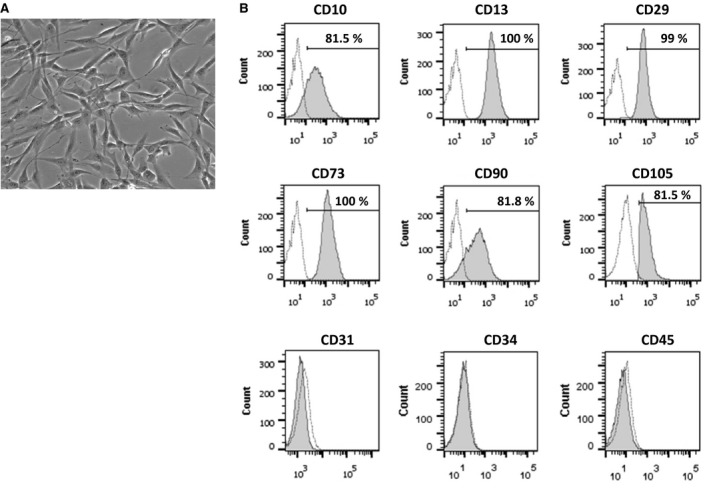
(**A**) Representative phase contrast image of ASC at passage 4 when cultured in EBM‐2mv media. (**B**) Fluorescence‐activated cell sorting analyses of ASC at passage 4. Fluorescent signals for cells with isotype IgG are shown as dotted line and antigen‐specific signals are shown as grey‐filled curves.

Studies involving human adipose tissue sample collection were approved by the Indiana University School of Medicine Institutional Review Board. Human subcutaneous adipose tissue samples obtained from lipoaspiration procedure were processed to isolate ASCs as described previously 29. In brief, the fat tissue was digested in collagenase type I solution (Worthington Biochemical, Lakewood, NJ, USA) under agitation for 1 hr at 37°C and centrifuged at 300 × *g* for 8 min. to separate the stromal cell fraction (pellet) from adipocytes. The pellet was re‐suspended in DMEM/F12 containing 10% FBS (Hyclone, Thermofisher.com) filtered through 100 μm Nitex filters (Sefar America Inc, Depew, NY, USA) and centrifuged at 300 × *g* for 8 min. The cell pellet was treated with red cell lysis buffer (154 mmol/l NH_4_Cl, 10 mmol/l KHCO_3_, 0.1 mmol/l EDTA) for 10 min. The final pellet was suspended in EGM2‐MV (Cambrex, East Rutherford, NJ, USA), plated and expanded. The cell purity and consistency in expression of previously described surface markers of ASC (CD10+, CD13+, CD29+, CD73+, CD105+, CD90) was confirmed by phase contrast microscopy and flow cytometric technique as described previously 30 and shown in Figure 1. Briefly, ASC at passage 4 was labelled with fluorescently tagged mouse anti‐human IgG for CD10, CD13, CD29, CD31, CD34, CD45, CD73 CD90 and CD105 or isotype control IgGs (all were purchased from BD Biosciences), ASC was negative for the haematopoietic marker CD45 and the endothelial marker CD31. *Passage‐four* cells were used for *in vivo* experiments.

### Surgical procedures and hASCs treatment

Ischaemia/reperfusion AKI or sham‐control surgery was conducted as described previously 2. Rats were anaesthetized with intraperitoneal injections of ketamine (100 mg/kg) with xylazine (5 mg/kg) and then placed on a heating pad to maintain physiological temperature. Bilateral renal ischaemia was induced by clamping both the right and left renal pedicle for 40 min. At the time of reperfusion, either control media or 2 × 10^6^ hASCs (in 0.5 ml of EBM‐2/1%BSA) were injected into the suprarenal aorta. Sham‐operated control animals (*n* = 4) were subjected to the same procedure without pedicle clamping. Animals were allowed to recover for 2 days (cohort 1: *n* = 10 for both experimental groups) or 7 days (cohort 2 *n* = 8 vehicle; *n* = 13 ASCs) to evaluate the effects of therapy on mitigation of early injury and tissue repair, respectively.

### Human ASC homing

hASCs (2 × 10^6^ hASCs in 0.5 ml of EBM‐2/1%BSA) were labelled using Celltracker CMTPX (Thermofisher.com) according to manufacture instructions and were injected into the suprarenal aorta at the time of reperfusion. Kidney, spleen, lung and hindlimb skeletal muscles were harvested at 10 min. and 48 hrs post‐injection, then immediately sectioned without fixation and evaluated for the presence of fluorescent cells using confocal microscopy (Olympus FV 1000‐MPE microscope, Center Valley, PA, USA).

### Measurement of renal function

At the indicated times, blood was collected in heparin‐treated Eppendorf tubes and centrifuged at 3000 × *g* for 10 min. Plasma creatinine was measured using creatinine reagent kit on Point Scientific QT 180 Analyzer (Point Scientific, Inc, Canton, MI, USA) according to manufacturer's specifications.

### Evaluation of renal structure

At the end of the study, kidneys were harvested for histological analysis and fluorescence‐activated cell sorting analysis. Portions of the kidney were fixed with either formalin or methanol. To evaluate the extent of renal tubular damage, formalin‐fixed paraffin‐embedded sections were stained with haematoxylin and eosin. Six random images (three cortex, three outer medulla) were obtained using a Leica DMLB (Scientific Instruments, Columbus, OH, USA) microscope using a 20× objective. For each kidney, an average of 60 tubules were scored from images by an observer who was blinded to the treatments using a 1–4 scoring system described previously 31. Data presented are based on the average score per tubule for each animal.

### Evaluation of microvessel density and myofibroblasts

Renal capillaries were stained as described previously 32. Briefly, methanol‐fixed vibratome sections of kidneys were subjected to immunofluorescent staining using the highly specific capillary marker, cablin (A generous gift from Dr. Robert Bacallao) 33. Cablin‐specific signals were developed using a tyramide signal amplification kit (Invitrogen, Carlsbad, CA, USA) as described previously 32. Confocal images were obtained using an Olympus FV 1000‐MPE microscope. Renal capillary density was determined from a minimum of 10 random images per kidney (five cortex, five outer medulla) by counting the number of stained structures intersecting an arbitrary grid, with the aid of ImageJ software (National Institutes of Health), as described 32. Data are expressed as % change in vessel density compared to the mean value of sham‐operated control rats.

In parallel, formalin‐fixed paraffin‐embedded kidney sections were stained with mouse anti‐rat alpha‐smooth muscle actin (α‐SMA) antibody (Invitrogen) and developed using Histomouse‐SP staining kit‐AEC (Life Technologies, Carlsbad, CA, USA). Three random images from renal outer medulla were obtained from each rat by light microscopy. Scoring of α‐SMA was quantified by overlaying an arbitrary array of gridlines to a density of 520 boxes per visual field with the aid of ImageJ software. The number of boxes containing α‐SMA‐positive structures was counted, and data are expressed as the % of boxes with α‐SMA‐positive structures per visual field.

### Fluorescence‐activated cell sorting

Harvested kidneys were minced and digested in TL Liberase (2 μg/ml; Roche) for 15 min. at 37°C in gentleMACS dissociator (Miltenyli, San Diego, CA, USA). The obtained cell suspension was filtered through a 100‐μm filter mesh and washed with medium. The lymphocytes were separated by Percoll (Sigma‐Aldrich, Carlsbad, CA, USA) and counted by haemocytometer 10. To label T lymphocytes, the cells were stained with anti‐rat antibodies against CD4 (PE‐Cy7), CD8a (Alexa 647), FOXp3 (PE) and CD25. To evaluate IL‐17 secretion by T cells, the cells were first stained for surface CD4, then permeabilized with 0.1% saponin and stained with antibodies against rat IL‐17 (FITC). Cells were scanned using a FACS Calibur analyzer (BD Biosciences, San Jose, CA, USA) and analysed using Flowjo software (Tree Star, Ashland, OR, USA). Gating strategies were exactly as described previously 10, and representative plots and associated gating strategies are shown in Figure S1.

### Statistical analysis

Data are presented as mean ± S.E.M, unless otherwise stated. Statistical analyses were performed with GraphPad Prism 5 (GraphPad Software, La Jolla, CA, USA). anova and Student's *t*‐test analysis were used to assess differences between data means as appropriate. Survival was evaluated using Log‐rank (Mantel‐Cox) test. A *P*‐value of <0.05 was used for the criterion of significance.

## Results

### Human ASCs significantly improves renal function following renal I/R

**Figure 2:**
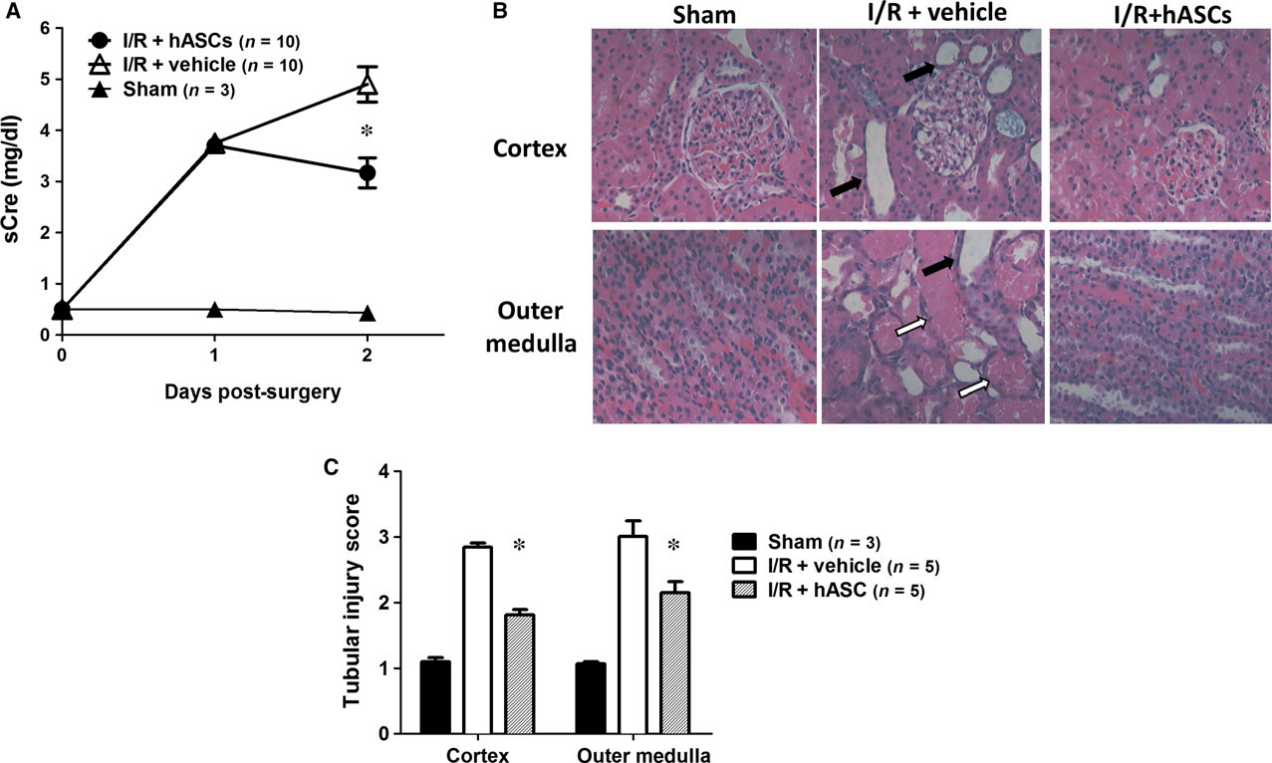
hASCs ameliorate renal injury following I/R. (**A**) Serum creatinine levels were measured in the rats 24 and 48 hrs post sham surgery or after I/R injury followed by infusion of 2 × 10^6^ hASC or vehicle. (**B**) Representative images of haematoxylin/eosin stained cross sections through renal cortex and outer medulla of kidneys harvested 48 hrs post sham‐operation or I/R followed by either vehicle or hASC treatment. Note the presence of dilated tubules containing sloughed cells (black arrow) and necrotic cellular debris (white arrow) in cortex and outer medulla of vehicle‐treated kidneys, while less severe tubular damage is observed in hASC‐treated kidneys. (**C**) Tubular injury scores derived from haematoxylin and eosin stained sections Data presented as mean ± S.E.M; *indicates *P* < 0.05 I/R+hASC *versus* I/R+vehicle by Student's *t*‐test.

To determine the effects of hASC on renal recovery following I/R in rat model, hASCs or vehicle was injected into the suprarenal aorta at the time of reperfusion and allowed to recover for 2 days (cohort 1). As the importance of inflammatory component in I/R injury is well described 10, 34, 35, 36, 37, this study was specifically designed with immunocompetent animals, rather than with immunocompromised athymic nude rats or NOD/SCID mice. It has been shown that systemic infusion of human mesenchymal cells, including ASC, into immunocompetent rodents and larger animals produce positive therapeutic effects 18, 22, 23, 38, 39, 40, without evident rejection responses. As expected, in vehicle‐treated rats, I/R resulted in a significant increase in serum creatinine at 24 and 48 hrs after injury/treatment (Fig. 2A). The loss of renal function was associated with a significant renal tubular damage with evidence of cellular necrosis, cell sloughing and tubular dilatation both in the renal cortex and renal medulla (Fig. 2B and C). When rats were treated with hASCs, serum creatinine levels were similar at 24 hrs post‐I/R relative to vehicle, but there was a significant diminution of its levels by 48 hrs (Fig. 2A) and the degree of tubular damage was also diminished at 48 hrs following I/R (Fig. 2B and C).

Previous studies that used bone marrow‐derived MSC in the setting of AKI have shown low frequency of homing of injected cells to the injured kidneys 41. To determine whether the therapeutic effect of human ASC depends on cell homing to the site of injury, we labelled ASC with Celltracker‐CMTPX (red fluorescent dye) and administered them at reperfusion. Confocal microscopy analysis of multiple cross sections of kidneys tissues, prepared immediately after harvest, has clearly shown that injected cells were undetectable in the kidneys neither at 10 min. nor at 48 hrs following renal I/R (Fig. S2). In contrast, a small number of cells were found in lung (Fig. S2) and potentially in skeletal muscles of hindlimbs (not shown), but not in spleen.

### hASCs infusion reduces infiltration of inflammatory cells following I/R

**Figure 3:**
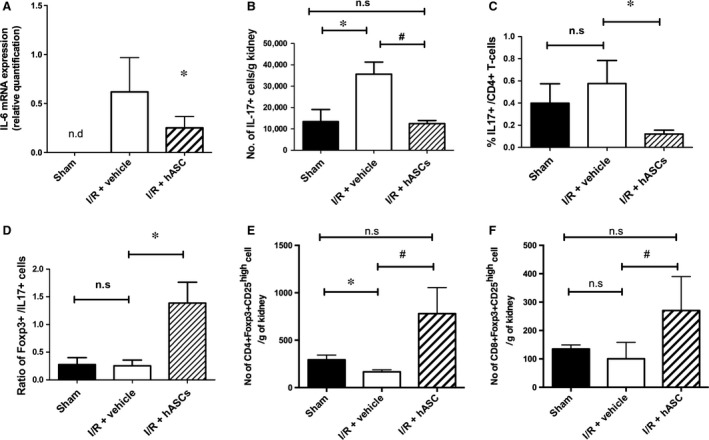
hASCs modulate inflammatory cytokines, reduce accumulation of IL17‐secreting cells and increase T regulatory cells in kidneys following I/R. (**A**) RT‐PCR of whole kidney RNA for IL‐6 is shown for post‐ischaemic *versus* ASC‐treated rat kidneys at 48 hrs post‐injury. (**B‐E**) Kidney resident lymphocytes were isolated from 48 hrs post‐surgery/treatment and evaluated for total number of IL17+ T cells (**B**), % of IL17+/CD4+ T cells (**C**) and the ratio of Foxp3+/IL17+ cells (**D**) and CD25high/Foxp3+ natural T regulatory cells (**E** and **F**) using fluorescence‐activated cell sorting. Data are expressed as mean ± S.E.M.; *indicates *P* < 0.05 I/R vehicle vs sham; # indicates *P* < 0.05 I/R+hASC vs I/R vehicle.

The increased expression of IL‐6 has been suggested to play an important role in mediating lymphocyte infiltration into post‐ischaemic kidney 42. Here we found that while renal I/R significantly increased renal IL‐6 expression, kidneys obtained from the rats that received ASC therapy had significantly attenuated expression of IL‐6 (Fig. 3A), thus suggesting that ASC convey anti‐inflammatory properties following renal injury.

Infiltrating cells including lymphocytes play a significant role in AKI 35, 36. When infiltrating lymphocytes were evaluated 48 hrs post‐I/R, the numbers of CD4+ and CD8+ lymphocytes and B cells were not affected by hASC treatment relative to vehicle (Table 1). Recently, Chan *et al*. and we previously demonstrated that T‐helper 17 (Th17) cells are increased early following ischaemia and contribute to the severity of AKI 10, 43. Consistent with our prior results, I/R injury significantly increased the total number of IL17+ expressing cells in the injured kidney relative to sham (Fig. 3B). Interestingly, hASC treatment protected kidneys from the accumulation of total IL17+ cells, as well as CD4 + IL17+ and CD8 + IL17+ when compared with vehicle‐treated I/R rats (Table 1). hASC therapy was accordingly associated with a decrease in the percentage of IL17 + /CD4+ T cells (Fig. 3C), but did not influence Th1 (CD4 + IL4+) or Th2 (CD4 + IFNγ+) polarization (Table 1). It has been suggested that T regulatory cells (Foxp3+) are associated with modulating T cell activity following renal injury 35. Interestingly, at 48 hrs post‐I/R, the number of CD4 + /Foxp3+ and CD8 + /Foxp3+ cells were significantly greater in hASC‐treated rats compared with vehicle (Table 1), which increased the ratio of regulatory Foxp3+ T cells to pro‐inflammatory IL17+ T cells (Fig. 3D). An additional study was conducted to determine whether the increase in T regulatory cells in ASC‐treated rats was due to the presence of CD25^high^/Foxp3+ population corresponding to natural T regulatory cells 44. Gating strategies to identify this population are shown in Figure S1. As shown in Figure 3E,F, ASC treatment significantly increased the number of CD25^high^/Foxp3 + CD4 or CD8 cells in 2‐day post‐ischaemic kidney relative to their levels in kidneys of vehicle‐treated rats. Taken together, these data indicate that hASC therapy modulates T cell differentiation post‐I/R by reducing IL17‐secreting cells and increasing the number of Foxp3+ T regulatory cells.

**Table 1:** Effect of hASCs on infiltration of cells following I/R. Shown are the number of different infiltrating cell types expressed per gram kidney weight 48 hrs following I/R. Data are expressed as mean ± S.E.M

	Treatment		
	Sham	I/R+ Vehicle	I/R+ hAsc
CD4+	6441 ± 100	8769 ± 1943	9420 ± 945
CD8+	7339 ± 756	19675 ± 5062	20055 ± 2761
B cells	13886 ± 620	10067 ± 1680	10403 ± 1438
DC/Macs	11030 ± 771	8603 ± 1176	9523 ± 1993
CD4 + IL‐4+ (Th1)	248 ± 22	251 ± 79	253 ± 44
CD4 + IFN‐γ+ (Th2)	408 ± 33	461 ± 60	408 ± 117
Total IL‐17+	13416 ± 5691	35697 ± 5600†	12509 ± 1446*
CD4 + IL‐17+	2573 ± 203	3615 ± 1000	1043 ± 281*
CD8 + IL‐17+	2082 ± 826	4146 ± 875	1722 ± 416*
CD4 + Foxp3+	852 ± 627	632 ± 148	1038 ± 159*
CD8 + Foxp3+	55 ± 12	137 ± 62	282 ± 81*

*indicates *P* < 0.05 I/R+hASC *versus* I/R+vehicle. ^†^indicates *P* < 0.05 in I/R+ vehicle *versus* sham by anova.

### hASCs improve survival and recovery following I/R

**Figure 4:**
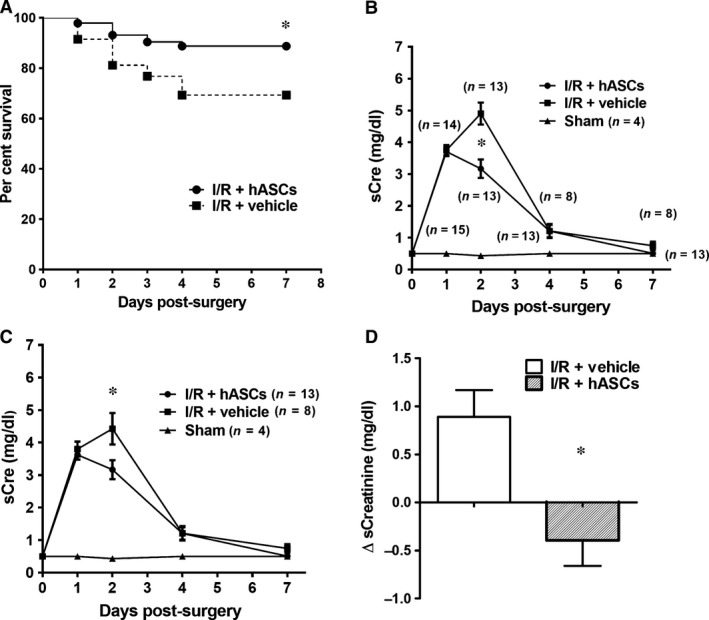
hASCs increase survival and enhance recovery following renal I/R. (**A**) Survival graph of post‐ischaemic rats treated with vehicle or hASCs. (* indicates *P* < 0.05 I/R+hASC *versus* I/R+vehicle by Mantel‐Cox test). (**B**) Serum creatinine values for sham‐operated, I/R+vehicle and I/R+hASCs are shown for all rats. Because of rat mortality over the course of the study, the ‘*n*’ for each treatment group is indicated in parentheses for each time‐point. (**C**) Serum creatinine values plotted only for rats that survived for the entire length of seven‐day experiment (rats that died during the experiment were excluded in this analysis). (**D**) Graph represents change in serum creatinine level between 24 and 48 hrs after injury/treatment. Data presented as mean ± S.E.M; *indicates *P* < 0.05 I/R+hASC *versus* I/R+vehicle by Student's *t*‐test.

Renal I/R injury results in a permanent loss of renal capillaries, which is detectable by 7 days post‐I/R, a time‐point at which improvement in renal function is evident 5, 45. Therefore, an additional study (cohort 2) was conducted by extending survival time to 7 days post‐I/R. Similar to cohort 1, serum creatinine levels were elevated at 24 and 48 hrs post‐I/R and were significantly reduced in hASC‐treated rats relative to vehicle‐treated rats at 48 hrs (Fig. 4B–D). hASC treatment substantially lowered the mortality rate of rats post‐I/R such that 31% of vehicle‐treated rats died by day 7 post‐I/R *versus* only 11% of hASC‐treated rats (*P* < 0.05) (Fig. 4A).

As is typical for this model, creatinine values return to sham‐operated control levels by day 7 in surviving vehicle‐ and hASC‐treated rats. Because of the effect on mortality, the number of animals contributing to each data point in Figure 4B is not consistent, but rather changes over the course of the study. Because animals that were most severely injured most likely perished during the recovery period, data on recovery of function (creatinine level) are biased by removing the contribution of the most severely injured rats. Therefore, we also examined serum creatinine levels using only data from rats that survived for the entire 7‐day protocol (Fig. 4C). Importantly, in surviving rats, the rise in serum creatinine at 24 hrs and the reduction at 48 hrs by hASCs was similar to that observed within the entire cohort (compare Fig. 4B to C).

**Figure 5:**
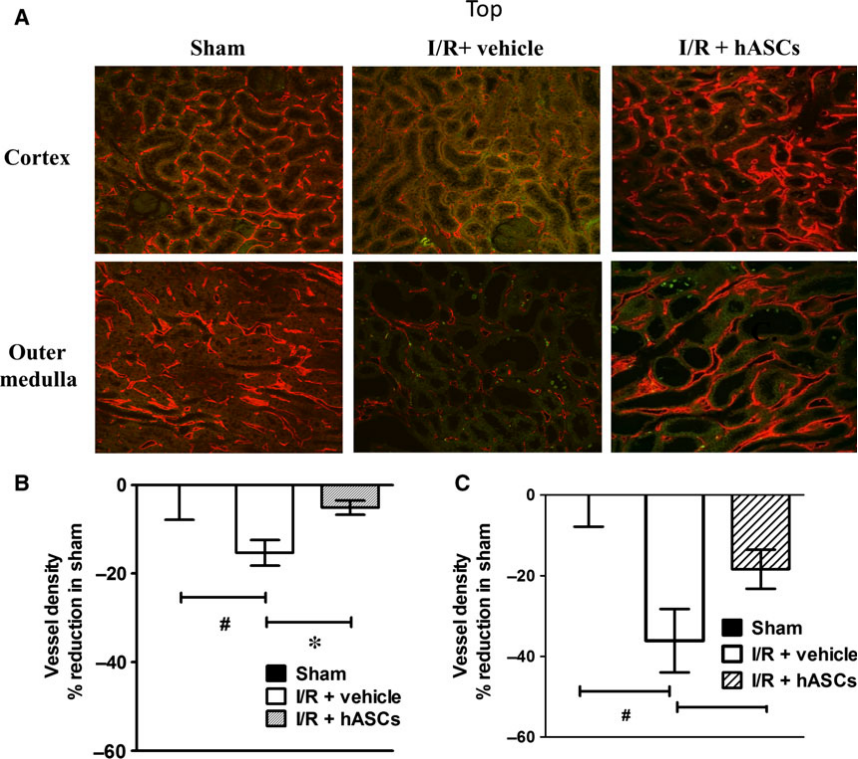
hASCs preserve renal capillary density following I/R. (**A**) Representative images of cablin‐stained cross sections of kidneys harvested 7 days following I/R injury/treatment or sham surgery. Confocal derived micrographs are shown for renal cortex or renal outer medulla. Cablin‐stained kidney sections were scored for vessel density using ImageJ and are shown for renal cortex (**B**) and outer medulla (**C**). Data are normalized to, and expressed as per cent of sham‐operated controls (mean ± S.E.M). *indicates *P* < 0.05 I/R+hASC *versus* I/R+vehicle; ^#^indicates *P* < 0.05 in I/R+ vehicle *versus* sham by anova.

Because ASCs have vasculogenic activities, we sought to determine whether hASCs would have beneficial effects on capillary preservation. Despite the resolution of serum creatinine by day 7, peritubular capillary density measured by cablin immunofluorescence was significantly reduced in vehicle‐treated I/R rats, consistent with previous observations 32. This reduction in capillary density was significantly attenuated in hASC‐treated rats both in the renal cortex (15% *versus* 5%) and outer medulla (36% *versus* 18%) (Fig. 5A–C).

### hASCs inhibit myofibroblast formation following I/R

**Figure 6:**
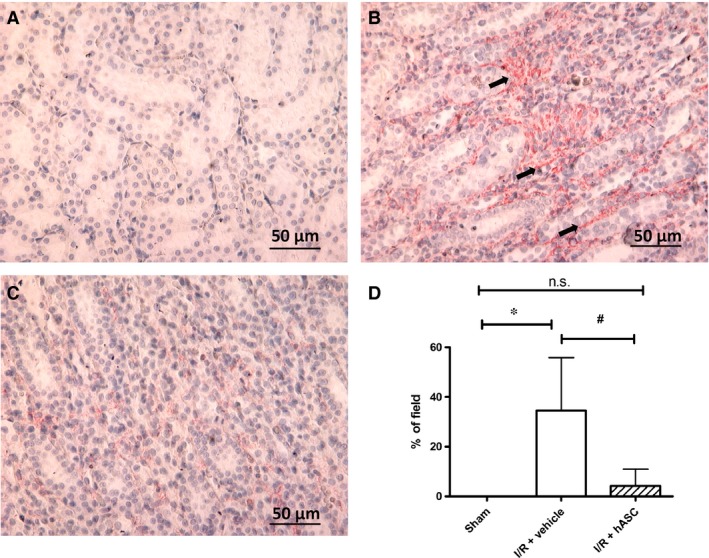
hASCs reduce myofibroblast formation following I/R injury. Representative images of α‐SMA stained sections of renal outer medulla (obtained 7 days following I/R) in (**A**) sham, (**B**) I/R+vehicle and (**C**) I/R+hASC kidneys. Black arrows in **B** indicate interstitial α‐SMA‐positive structures (**D)** Data are expressed as the % of defined areas per visual field containing α‐SMA‐positive structures; *indicates *P* < 0.05 sham *versus* I/R+vehicle; ^#^indicates *P* < 0.05 I/R+hASC *versus* I/R+vehicle by anova. n.s – not significant.

The activity of αSMA‐positive myofibroblasts is considered to represent an important mediator of renal interstitial fibrosis. Therefore, we also evaluated the effect of hASC on myofibroblast content 7 days following I/R. As expected, vehicle‐treated rats demonstrated elevated myofibroblast staining compared to sham‐operated controls (Fig. 6A/B), while hASC‐treated rats showed a dramatic reduction in myofibroblast staining relative to vehicle‐treated rats (Fig. 6C/D).

## Discussion

Acute kidney injury remains a significant problem in the clinical setting. Despite advances in renal replacement therapies, mortality rates have remained at high levels for decades, with no improvement 1, 46. Moreover, surviving patients are predisposed to develop CKD 45. There is therefore a pressing need for novel therapies to promote renal cellular repair and improve tissue remodelling. Over the past decade, advances in the field of regenerative medicine allowed the development of cell‐based therapies that can potentially benefit kidney repair. Previous studies have found that bone marrow‐derived MSCs have reno‐protective effects and enhance tissue regeneration after AKI in rodents 12, 13, 26, 47, 48, 49, 50, suggesting that such cell therapy holds potential as an option to treat patients with AKI. ASCs, which share many properties of bone marrow MSCs and are as potent in multiple regenerative applications, have gained significant interest as a clinically feasible therapeutic option. ASCs, which are abundant in the adult human body, are known to produce a variety of angiogenic, pro‐survival and immunomodulatory factors 51. It has been demonstrated that ASCs, through both paracrine and direct physical interaction with endothelial cells, modulate angiogenesis and vascular stability 19, 29, 52.

Primary characteristics of renal I/R injury are the increase in serum creatinine and damage to the tubular epithelium 2. Bi *et al*. demonstrated that mouse ASCs diminished renal tubular apoptosis, preserved renal function and improved survival in cisplatin‐induced AKI 26. Recently, Katsuno *et al*. demonstrated the potential efficacy of hASC to ameliorate injury in a folate model of AKI in rats 53. The current study extends these observations by demonstrating the potential for human ASCs to ameliorate renal tubular damage and limit both inflammation and subsequent deterioration of the microvasculature following I/R injury. The reduction in serum creatinine was not immediate (24 hrs post‐I/R) but evident by 48 hrs, similar to previous observations using MSCs 13. However, in contrast to other studies using MSCs which did not show a significant effect on mortality 13, our current study using ASCs showed a significant reduction in mortality in this model of AKI. The exact mechanisms by which hASCs convey protection and reduce renal injury is unclear and likely multifactorial. Several parallel studies have failed to demonstrate MSC retention 26, 29, 53 after infusion, whereas other studies conducted with MSCs and ASCs suggested that the primary therapeutic effects are due to secreted factors. In support of prior studies, our analysis of multiple section of the kidneys has clearly shown minimal, if any, homing and retention of Celltracker‐CMTPX‐labelled hASCs in the treated kidneys. This supports the hypothesis that therapeutic effects of ASC are mediated by paracrine activity at a distance 54. Further studies will be conducted to specifically assess the reno‐ and vascular protective effects of concentrated media conditioned by ASC.

Inflammatory cell infiltration plays a prominent role in the evolution of AKI, primarily in the outer medulla 2. Following I/R, the injured renal parenchymal cells and endothelial cells facilitate trafficking of innate lymphoid cells into the kidney 55. T cells, depending upon the activating signals, can differentiate into several effector T‐helper subsets. Recently, it has been suggested that Th17 cells play a role in kidney injury, as IL17A‐deficient mice, manifest reduced injury in a model of cisplatin‐induced AKI 43. We have previously shown that Th17 cells play an important role in AKI and subsequent progression to CKD 10. Multiple studies suggest that MSCs modulate Th17 polarization *in vitro* 56, 57. Furthermore, in an experimental autoimmune encephalomyelitis (EAE) mouse model, treatment with MSCs reduced Th1 and Th17 differentiation and increased T regulatory cells 58. In parallel with these results, the current study indicates for the first time that hASCs do not influence the accumulation of CD4+ and CD8+ T cells in the kidney, but rather inhibit their polarization into a pro‐inflammatory (Th17+) phenotype 53 (Table 1). hASCs further modulate the inflammatory response by increasing T regulatory (Foxp3+) cells in kidney parenchyma.

A common feature present in all models of kidney disease is the reduction in peritubular capillary density, which is thought to fuel hypoxia and accelerate the development of interstitial fibrosis 5, 59. The exacerbation of renal hypoxia is known to stimulate the production of pro‐fibrotic factors such as TGF‐β and extracellular matrix molecules such as collagen and fibronectin, further expanding the interstitial compartment and leading to a decrease in the efficiency of oxygen delivery to the tubules, creating a vicious cycle for progressive renal disease 5. Thus, loss of peritubular capillaries and the generation of renal interstitial cells that produce extracellular matrix are of central interest in understanding the development of renal fibrosis. With regard to the loss of endothelial cells, we have demonstrated that endothelial to mesenchymal cell transition (EndoMT) plays an important role contributing to capillary rarefaction 32. This activity can be reversed by the exogenous administration of VEGF‐121 32. It has also been suggested that pericyte activation, in the setting of renal ischaemia, results from a loss of contact with injured peritubular capillary endothelial cells. Elegant studies have demonstrated that injury‐activated pericytes lose their ability to stabilize capillary endothelial cells and are a primary source of interstitial myofibroblasts contributing to renal fibrosis 60, 61, 62. In the current study, hASCs, which are known to have pro‐angiogenic properties, were shown to preserve capillaries and attenuate myofibroblast development following I/R, features thought to be critical for the progression of CKD. We have shown that ASC secrete multiple endothelial survival factors, including VEGF and HGF as well as SDF‐1, the factors responsible for attaching circulating bone marrow‐derived progenitor cells to the site of injury 17, 63, 64. Whether the primary targets of ASC therapy are endothelial cells, pericytes or lymphocytes, or whether ASC provide their effects through several parallel mechanisms in regard to the development of interstitial fibrosis is not yet clear.

In summary, we have demonstrated that hASCs convey vascular protection, decrease inflammation and incite significant repair following AKI in immunocompetent rats. Recently, potentially promising studies from a phase 1 clinical trial indicated the potential benefit of bone marrow‐derived MSCs on deterioration of renal function, hospital length of stay and need for readmission in a population of coronary artery bypass patients susceptible to AKI 65. However, these studies were not focused on long‐term renal functional end‐points, and no further follow‐up trials have been published. Given that AKI is associated with CKD development, a growing challenge in patient care will be developing therapeutic strategies to minimize progression following AKI. In addition to improving acute effects and survival, our data suggest a potential long‐term benefit as the primary mediators of CKD, that is vascular rarefaction, myofibroblast formation and inflammation, are attenuated with hASC treatment. Given the increased momentum of ASCs in clinical trials, we propose that these cells may be useful for clinical application in AKI with end‐points focused on both recovery and attenuation of CKD.

## Disclosure of potential conflict of interests

None.

## Authors’ contribution

JAC performed the conception and design, collection and assembly of data, data analysis and interpretation, manuscript writing. DOT performed the conception and design, provision of study material, data analysis and interpretation, final approval of manuscript. PM performed the collection and/or assembly of data, data analysis and interpretation, final approval of manuscript. AC performed the collection and/or assembly of data, data analysis and interpretation, final approval of manuscript. SM‐C performed the provision of study material. KLM performed the conception and design, financial support, administrative support, data analysis and interpretation, final approval of manuscript. DPB performed the conception and design, financial support, data analysis and interpretation, manuscript writing and final approval of manuscript.

## Supporting information


**Figure S1** Gating strategies for the phenotypic analysis of infiltrating CD4+ T cells in the kidney. Lymphocytes were gated based on the forward scatter *versus* side scatter, which is further gated on CD4+/CD8+ T cells. Right column: These T cells were analyzed further based on the activation markers for IL‐17 (shown as histogram) or IL4 and IFNγ (shown as dot plot). For T‐regulatory cells, the population of T‐regulatory cells was defined as Foxp3+/CD25^high^ using contour plot analysis as shown in the lower right panel.

**Figure S2** hASC fail to home to the kidney following ischemia reperfusion injury. Shown are representative confocal images of Celltracker‐CMTPX labeled ASCs *in vitro* just prior to injection (**A**) and in kidney (**B** and **D**) or lung (**C** and **E**) at 10 min. (**B** and **C**) or 48 hrs (**D** and **E**) following administration. No cells were detected in kidney at any time, while cells were occasionally observed in lung. Representative of 3 animals per time point.
