# Supplementary figures and images for: Human adipose stromal cell therapy improves survival and reduces renal inflammation and capillary rarefaction in acute kidney injury
Source: J Cell Mol Med. 2017 Apr 28;21(7):1420–30. doi: 10.1111/jcmm.13071 (PMC5487924; doi:10.1111/jcmm.13071)

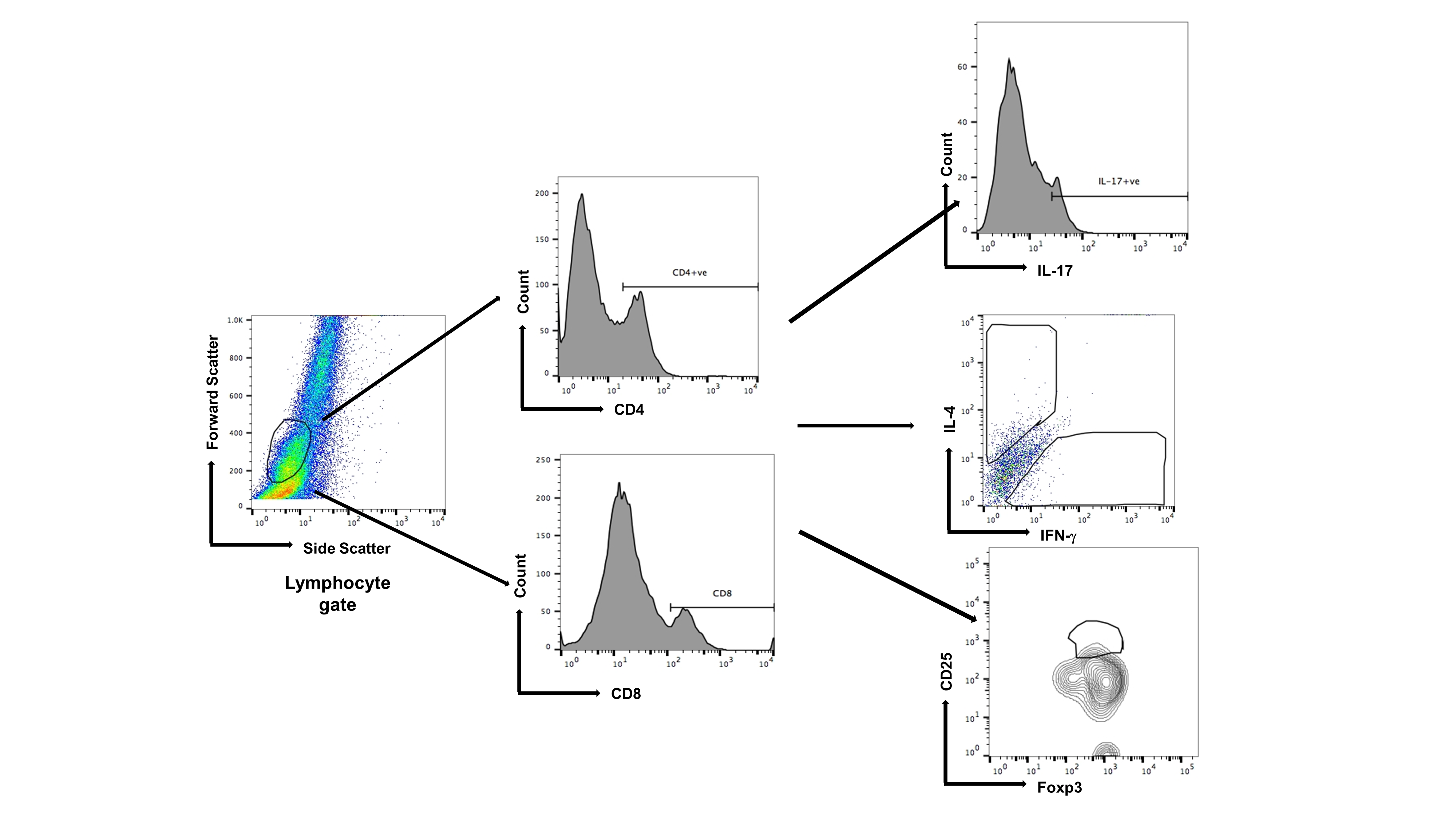

Supplement: Supplementary file 1 — Figure S1 Gating strategies for the phenotypic analysis of infiltrating CD4+ T cells in the kidney. Lymphocytes were gated based on the forward scatter versus side scatter, which is further gated on CD4+/CD8+ T cells. Right column: These T cells were analyzed further based on the activation markers for IL‐17 (shown as histogram) or IL4 and IFNγ (shown as dot plot). For T‐regulatory cells, the population of T‐regulatory cells was defined as Foxp3+/CD25high using contour plot analysis as shown in the lower right panel. [file JCMM-21-1420-s001.TIF]

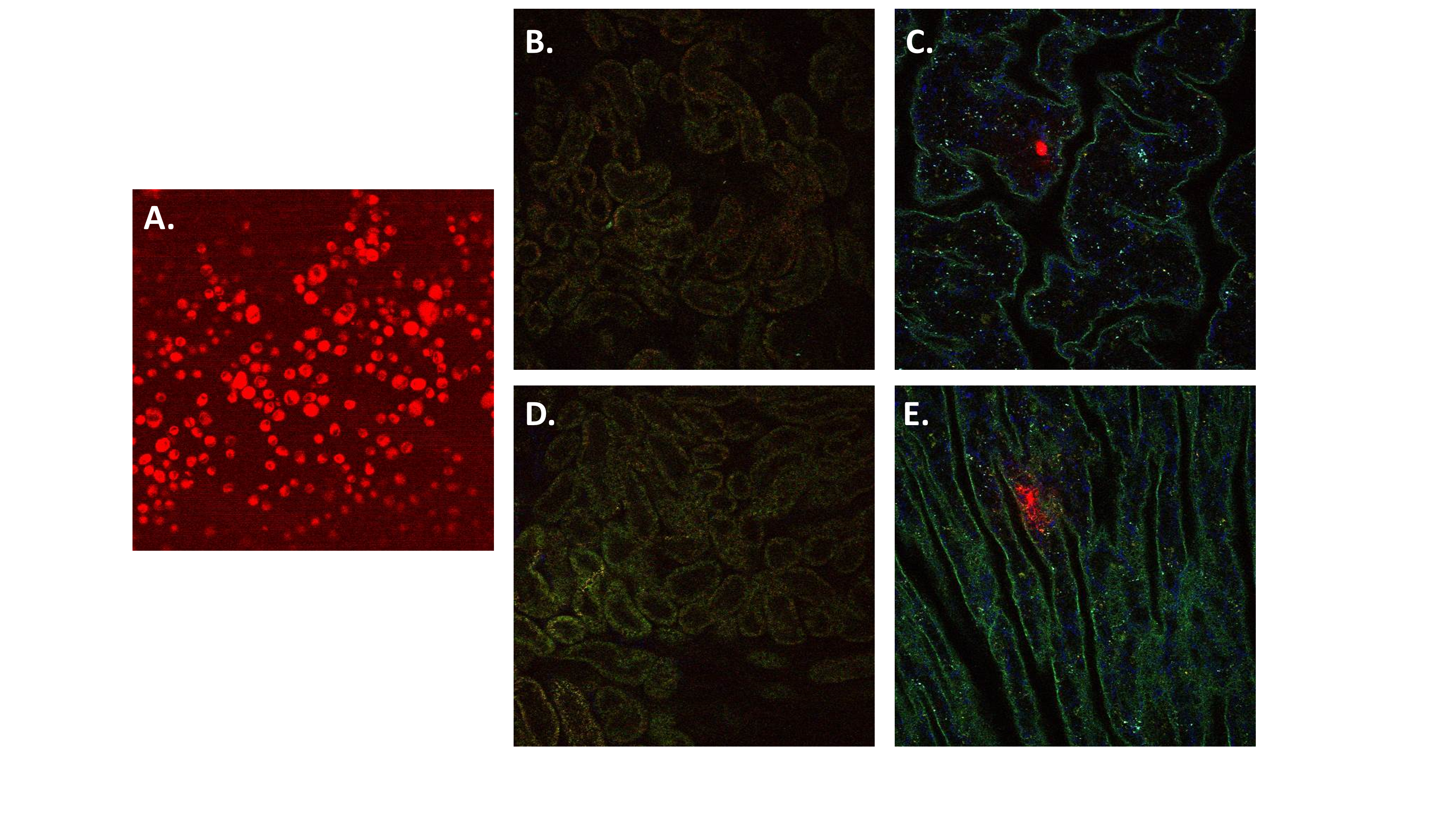

Supplement: Supplementary file 2 — Figure S2 hASC fail to home to the kidney following ischemia reperfusion injury. Shown are representative confocal images of Celltracker‐CMTPX labeled ASCs in vitro just prior to injection (A) and in kidney (B and D) or lung (C and E) at 10 min. (B and C) or 48 hrs (D and E) following administration. No cells were detected in kidney at any time, while cells were occasionally observed in lung. Representative of 3 animals per time point. [file JCMM-21-1420-s002.TIF]
